# The open targets post-GWAS analysis pipeline

**DOI:** 10.1093/bioinformatics/btaa020

**Published:** 2020-01-13

**Authors:** Gareth Peat, William Jones, Michael Nuhn, José Carlos Marugán, William Newell, Ian Dunham, Daniel Zerbino

**Affiliations:** b1 European Molecular Biology Laboratory, European Bioinformatics Institute, Wellcome Genome Campus, Hinxton, Cambridge CB10 1SD, UK; b2 Open Targets, EBI South Building, Wellcome Genome Campus, Hinxton, Cambridge CB10 1SD, UK; b3 Wellcome Sanger Institute, Wellcome Genome Campus, Hinxton, Cambridge CB10 1SD, UK; b4 GSK, Medicines Research Center, Stevenage SG1 2NY, UK

## Abstract

**Motivation:**

Genome-wide association studies (GWAS) are a powerful method to detect even weak associations between variants and phenotypes; however, many of the identified associated variants are in non-coding regions, and presumably influence gene expression regulation. Identifying potential drug targets, i.e. causal protein-coding genes, therefore, requires crossing the genetics results with functional data.

**Results:**

We present a novel data integration pipeline that analyses GWAS results in the light of experimental epigenetic and *cis*-regulatory datasets, such as ChIP-Seq, Promoter-Capture Hi-C or eQTL, and presents them in a single report, which can be used for inferring likely causal genes. This pipeline was then fed into an interactive data resource.

**Availability and implementation:**

The analysis code is available at www.github.com/Ensembl/postgap and the interactive data browser at postgwas.opentargets.io.

## 1 Introduction

Genome-wide association studies (GWAS) are a powerful method to analyse common diseases in a large cohort. Taking advantage of affordable large-scale genotyping chip technologies, such studies are now routinely run across cohorts large enough to detect even weak associations between common variants and a phenotype of interest. There are now enough GWAS studies to warrant the existence of specialized databases, such as the GWAS Catalog (MacArthur *et al.*, 2017).

Despite this wealth of data, GWAS have not succeeded in translating into many therapeutic success stories (Huang, 2015). The main bottleneck is inferring truly causal genes from the GWAS results that can then be used as drug targets and thus new therapies. This gap between genetics research and translational applications is largely explained by the difficulty in functionally interpreting non-coding variants. Although annotating and prioritizing coding variants are a well-studied problem, determining the regulatory effect of non-coding variants is still difficult. In effect, many of the drug targets tested by the pharmaceutical industry fail to yield a new drug because they are revealed to be unrelated to the phenotype (Cook *et al.*, 2014).

To close this gap, a number of experimental techniques have been developed, such as molecular Quantitative Trait Loci (QTL) (Brem *et al.*, 2002), covariance analysis in chromatin state between distant regions of the genome (Thurman *et al.*, 2012) or sequencing-based assays, such as Promoter-Capture Hi-C (Javierre *et al.*, 2016). Existing pipelines (Shen *et al.*, 2017) integrate all these datasets but they do not connect directly to databases to gather their latest results.

We present here a pipeline that compares GWAS results to a collection of useful *cis-*regulatory datasets. We have run our pipeline across all GWAS Catalog studies and present the results in an interactive web interface, which can be used to examine the evidence supporting the candidate causal genes.

## 2 Methods

The analysis automates a number of standard post-GWAS data integration steps as follows:

1. (Optional) Search through public GWAS databases: GWAS Catalog, GRASP (Leslie *et al.*, 2014) or PheWAS Catalog (Denny *et al.*, 2013), using ontology terms where possible [using the EMBL-EBI Zooma (https://www.ebi.ac.uk/spot/zooma) term suggestion service to map text where possible to ontology terms].
2. Linkage disequilibrium (LD) expansion. By default, the 1000 Genomes genotypes (The 1000 Genomes Project Consortium, 2015) are used; however, it is possible to replace them with other cohorts, simply by replacing VCF files. Any significant single nucleotide polymorphism (SNP) is connected to nearby common SNPs with a Pearson *r*^2^ correlation >0.7.
3. Clustering. Each significant SNP and its LD neighbours form a cluster. Overlapping clusters in the same study are merged.
4. Alignment to known regulatory annotations, indicative of whether an SNP has any regulatory effect, in particular, the Ensembl Regulatory Build (Zerbino *et al.*, 2015) and RegulomeDB (Boyle et al., 2012).
5. Alignment to known *cis*-regulatory annotations, indicative of whether an SNP regulates a specific gene, in particular, GTEx (GTEx Consortium, 2017), Ensembl VEP (McLaren *et al.*, 2016), Fantom5 (Andersson *et al.*, 2014), ENCODE DNAseI hypersensitivity correlations (Thurman et al., 2012) and Promoter-Capture Hi-C calls (Javierre *et al.*, 2016), each evidence being assigned a weight between 0 and 1.
6. Computing an aggregate score for each (SNP, Gene) pair by summation of all the scores obtained in steps (4) and (5).

### 2.1 A deployable pipeline

The pipeline is coded in Python and was designed to be easily installed locally and run privately, whether against public databases or on a private dataset (provided as summary statistics in a tab-delimited file).

### 2.2 An interactive website

**Fig. 1.**
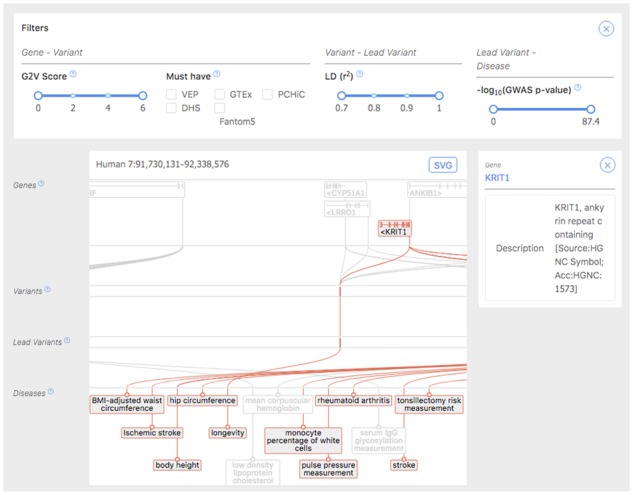
Screenshot of the post-GWAS browser. At the top, dynamic filters control what is displayed. Below, a genomic browser represents a genomic locus with genes, which are connected to nearby regulatory variants, which are in turn in LD with tag SNPs, associated with given phenotypes

The Open Target post-GWAS web browser allows users to browse through the pre-computed results of the pipeline run across all of GWAS Catalog. If searching from an SNP rsID or a gene symbol the browser displays either a genomic view or a table of associations (see Fig. 1). If searching for a disease, a table of known associations are displayed.

## 3 Results

We ran our pipeline on all GWAS Catalog studies at the time (last update December 7, 2018). This comprised 2092 phenotypes and diseases, described in 3187 publications, and a total of 67 771 significant SNPs. After LD expansion, a total of 923 891 unique SNPs were analysed, each SNP being involved in 290 publications on average. The average run time for each study was 40 min.

## 4 Conclusions

GWAS is a powerful approach to understanding disease mechanism but requires functional analysis to produce actionable results. The Open Targets post-GWAS pipeline facilitates this process, both through an automated tool and pre-processed results, freeing GWAS analysts from the laborious process of data integration.

## Funding

This work was supported by the Open Targets project and EMBL core funds to D.R.Z. and W.J.

*Conflict of Interest*: none declared.
